# The combination of elbasvir and grazoprevir for the treatment of chronic HCV infection in Japanese patients: a randomized phase II/III study

**DOI:** 10.1007/s00535-016-1285-y

**Published:** 2016-11-21

**Authors:** Hiromitsu Kumada, Yoshiyuki Suzuki, Yoshiyasu Karino, Kazuaki Chayama, Norifumi Kawada, Takeshi Okanoue, Yoshito Itoh, Satoshi Mochida, Hidenori Toyoda, Hitoshi Yoshiji, Shintaro Takaki, Naoyoshi Yatsuzuka, Etsuo Yodoya, Takashi Iwasa, Go Fujimoto, Michael N. Robertson, Stuart Black, Luzelena Caro, Janice Wahl

**Affiliations:** 1 Department of Hepatology, Toranomon Hospital, 1-3-1 Kajigaya, Takatsu-ku, Kawasaki, Kanagawa 213-8587 Japan; 2 Department of Gastroenterology, Sapporo Kosei General Hospital, North-3 East-8, Chuou-ku, Sapporo, Hokkaido 060-0033 Japan; 3 Department of Gastroenterology and Metabolism, Hiroshima University, 1-2-3 Kasumi, Minami-ku, Hiroshima, 734-8551 Japan; 4 Department of Hepatology, Osaka City University Medical School, 1-4-3 Asahimachi, Abeno-ku, Osaka, 545-8585 Japan; 5 Department of Gastroenterology and Hepatology, Saiseikai Suita Hospital, 1-2 Kawazono-cho, Suita-Shi, Osaka, 564-0013 Japan; 6 Department of Molecular Gastroenterology and Hepatology, Kyoto Prefectural University of Medicine, 465 Kajii-cho, Kawaramachi Hirokoji, Kamigyo-ku, Kyoto, 602-8566 Japan; 7 Department of Gastroenterology and Hepatology, Saitama Medical University, 38 Morohongo, Moroyama-machi, Iruma-gun, Saitama, 350-0495 Japan; 8 Department of Gastroenterology, Ogaki Municipal Hospital, 4-86 Minaminokawa, Ogaki, 503-8502 Japan; 9 Third Department of Internal Medicine, Nara Medical University, 840 Shijo-cho, Kashihara, Nara 634-8522 Japan; 10 Department of Gastroenterology/Liver Center, Hiroshima Red Cross Hospital and Atomic-Bomb Survivors Hospital, 1-9-6, Senda-machi, Naka-ku, Hiroshima, 730-8619 Japan; 11 MSD K.K., 1-13-12 Kudan-kita, Chiyoda-ku, Tokyo, 102-8667 Japan; 12 Merck & Co.Inc., 2000 Galloping Hill Road, Kenilworth, NJ 07033 USA

**Keywords:** Clinical trial, Therapy, Genotype, Sustained virologic response, Efficacy

## Abstract

**Background:**

Elbasvir (EBR) in combination with grazoprevir (GZR) has demonstrated efficacy in patients with hepatitis C virus (HCV) infections in trials primarily conducted in the USA and Europe. We investigated the safety and efficacy of EBR in combination with GZR in Japanese patients with chronic HCV infection, with or without cirrhosis.

**Methods:**

The study was conducted in two parts. In part 1, noncirrhotic patients were randomized 1:1 to receive EBR (50 mg) in combination with GZR (50 or 100 mg) once daily for 12 weeks. In part 2, noncirrhotic patients were randomized 3:1 to receive immediate or deferred treatment with EBR (50 mg) and GZR (100 mg, determined in part 1) for 12 weeks; cirrhotic patients received open-label immediate treatment. The primary efficacy end point was the rate of sustained virologic response 12 weeks after completion of the study treatment.

**Results:**

In part 1, 63 patients were randomized to receive EBR in combination with GZR at a dose of 50 mg (*n* = 31) or 100 mg (*n* = 32). The SVR12 rates were 100% with GZR at a dose of 50 mg and 96.8% with GZR at a dose of 100 mg. Tolerability was similar in both arms. In part 2, 301 noncirrhotic patients were randomized to receive immediate treatment (*n* = 227) or deferred treatment (*n* = 74), and 35 cirrhotic patients were enrolled. The SVR12 rates were 96.5% and 97.1% after immediate treatment in noncirrhotic and cirrhotic patients respectively. Safety was generally similar between immediate and deferred treatment.

**Conclusion:**

Treatment with EBR in combination with GZR for 12 weeks is effective and well tolerated in Japanese patients with chronic HCV infection.

**ClinicalTrials.gov identifier:**

NCT02203149.

**Electronic supplementary material:**

The online version of this article (doi:10.1007/s00535-016-1285-y) contains supplementary material, which is available to authorized users.

## Introduction

Hepatitis C virus (HCV) infection is a significant health care burden in Japan. The estimated adult prevalence of HCV infection is 1.5% of the Japanese population, amounting to approximately 1.6 million infected individuals [1]. Seventy percent of cases of hepatocellular carcinoma in Japan are attributable to HCV infection, and it is the fourth leading cause of death in men and the fifth leading cause of death in women [2]. The virus spread rapidly among intravenous drug users and through medical procedures (such as blood transfusions and contaminated syringes) around the time of World War II; as a result, the prevalence today is higher among the older generation than in younger people (more than 2% vs 0.1–0.2%) [3, 4]. Consequently, treatment algorithms focus on treatment outcomes in elderly patients [5]. Direct-acting antiviral agents have dramatically altered the treatment landscape for patients with chronic HCV infection in Japan in the last 5 years. The treatment options for patients with HCV genotype (GT) 1b infection include ledipasvir plus sofosbuvir and ombitasvir plus paritaprevir plus ritonavir, with daclatasvir plus asunaprevir also an option in patients if the Y93 or L31 variants are not present. Simeprevir or vaniprevir in combination with peginterferon and ribavirin are options for patients eligible for interferon (IFN)-based treatment [5].

The combination of elbasvir (EBR), an NS5A inhibitor, and grazoprevir (GZR), an NS3/4a inhibitor, has been recently approved by the US Food and Drug Administration for the treatment of chronic HCV GT1 and GT4 infection [6]. This combination therapy has potent antiviral activity in vitro [7, 8] and has demonstrated high efficacy in phase II and phase III clinical trials across a wide spectrum of patients with HCV infection, including those with cirrhosis, chronic kidney disease, or HIV coinfection, or in whom both IFN-based and peginterferon plus ribavirin plus NS3/4a protease inhibitor therapy previously failed [9–15]. Rates of sustained virologic response (SVR) greater than 90% have been achieved with 12-week regimens of EBR plus GZR in phase III studies conducted primarily in the USA and Europe. In addition, the use of a deferred-treatment group in the phase III C-EDGE Treatment Naive trial showed that EBR plus GZR has a similar safety profile in cirrhotic and noncirrhotic patients, and in active treatment and placebo-treated patients [11].

The objectives of the current phase II and phase III studies were to determine the safety and efficacy of once daily oral administration of EBR plus GZR when administered for 12 weeks in Japanese patients with chronic HCV infection, with or without cirrhosis. The phase II component of the study evaluated two dosages of GZR (50 or 100 mg/day) in combination with EBR (50 mg/day); the phase III study evaluated the preferred regimen from the phase II study in a larger randomized population, and incorporated a deferred active treatment group to provide a placebo-based comparative assessment of safety.

## Methods

### Patients

Japanese male or female patients aged 20–80 years with chronic HCV GT1 infection (HCV RNA level 100,000 IU/mL or greater in peripheral blood), with or without compensated cirrhosis, were enrolled. Chronic HCV infection was defined as the presence of anti-HCV antibody or RNA at least 6 months before enrollment or positive anti-HCV antibody or RNA with liver biopsy findings consistent with chronic HCV infection. Cirrhosis was defined as METAVIR stage 4 fibrosis on liver biopsy before day 1 or FibroScan stiffness greater than 12.5 kPa within 12 months before enrollment. The patients were treatment naïve (including those ineligible for IFN-based treatment), intolerant to IFN-based treatments, or treatment experienced (defined as relapse, breakthrough, or partial or null response to prior IFN-based therapy).

Patients with decompensated liver disease, hepatitis B virus or HIV coinfection, a history of malignancy, evidence of hepatocellular carcinoma, a history of gastric surgery or malabsorption disorders, or a history of chronic hepatitis not caused by HCV were excluded. Prestudy laboratory abnormality exclusion criteria included a hemoglobin level less than 9.5 g/L, creatinine clearance rate less than 50 mL/min, platelet count less than 50 × 10^3^/µL, serum albumin level less than 3.0 g/dL, and aminotransferase levels greater than ten times the upper limit of normal (ULN).

### Study design

The study was conducted in two parts. The aim of part 1 was to determine the optimal dose of GZR for use in combination with EBR for further assessment in part 2 of the study. Part 1 was a phase II multicenter, double-blind trial in which noncirrhotic patients were randomized 1:1 to receive EBR (50 mg) in combination with GZR (50 or 100 mg) once daily for 12 weeks. Patients randomized to receive GZR at a dose of 100 mg received two 50-mg tablets once daily; patients randomized to receive GZR at a dose of 50 mg received one 50-mg tablet once daily plus a matching placebo tablet. GZR and placebo were packaged identically to ensure maintenance of blinding. All patients were followed up for 24 weeks after treatment. A subgroup of 29 patients (14 patients who received GZR at a dose of 50 mg and 15 patients who received GZR at a dose of 100 mg) were enrolled in an intensive pharmacokinetic cohort.

Part 2 was a phase III, randomized, multicenter, double-blind, placebo-controlled trial to assess the safety and efficacy of orally administered EBR at a dose of 50 mg in combination with orally administered GZR at a dose of 100 mg (the dose selected from part 1). Noncirrhotic patients were randomized in a 3:1 ratio to receive EBR plus GZR for 12 weeks (immediate-treatment group) or placebo for 12 weeks followed by deferred active treatment with EBR plus GZR for 12 weeks (deferred-treatment group). The same blinding technique used in part 1 of the study (identically packaged matching placebos) was used for part 2. The study was unblinded after 4 weeks of follow-up, and thereafter active therapy was initiated in patients in the deferred-treatment group. Cirrhotic patients were allocated to a separate treatment arm, where they received open-label orally administered EBR (50 mg) plus orally administered GZR (either 50 or 100 mg as determined in part 1) once daily for 12 weeks.

Randomization was performed according to a computer-generated allocation schedule. In part 1, randomization was stratified by age (younger than 65 years vs 65 years or older). In part 2, randomization was stratified by age (younger than 65 years vs 65 years or older) and prior treatment experience (naïve vs intolerant vs prior relapse vs prior nonresponse). All patients were followed up for 24 weeks after completion of all study therapy.

The study was conducted in accordance with the provisions of the Declaration of Helsinki, the International Council for Harmonisation of Technical Requirements for Pharmaceuticals for Human Use guidelines, and other regulations governing clinical study conduct. The protocol was approved by an independent ethics committee or institutional review board at each participating site. All patients provided written informed consent. The study was registered at ClinicalTrials.gov (NCT02203149; protocol number PN058), and the protocol is available in the electronic supplementary material.

### Outcomes

The primary end point for part 1 was safety and tolerability of EBR at a dose of 50 mg in combination with GZR at a dose of 50 or 100 mg. For part 2, the primary end points were the SVR12 rate, defined as the proportion of patients with undetectable HCV RNA (target not detected) at 12 weeks after completion of all study treatment, and the safety and tolerability of EBR at a dose of 50 mg in combination with GZR at the selected dose. The secondary end points in parts 1 and 2 included the proportion of patients with HCV RNA target not detected in treatment week 2 (very early rapid viral response), in treatment week 4 (rapid viral response), at the end of treatment, 4 weeks after the end of all study treatment (SVR4), and 24 weeks after the end of all study treatment (SVR24), and the proportion of patients achieving HCV RNA levels less than 15 IU/mL (either target not detected or target detected but unquantifiable) at each time point, including at 12 weeks after the end of all study treatment. The exploratory end points included pharmacokinetics of EBR and GZR [area under the curve (AUC), maximum drug concentration (*C*_max_), drug concentration immediately before the next dose (*C*_trough_), and time to occurrence of maximum drug concentration (*T*_max_)], emergence of viral mutations resistant to EBR and GZR (parts 1 and 2), and the efficacy, safety, and tolerability of EBR and GZR in patients with cirrhosis (part 2 only).

This article includes data to follow-up week 12 for patients in the immediate-treatment group and to follow-up week 4 for patients in the deferred-treatment group. SVR24 data from the immediate-treatment group and active treatment in the deferred-treatment group will be reported elsewhere.

### Assessments

HCV RNA was measured by quantitative reverse transcription polymerase chain reaction (cobas^®^ TaqMan^®^ HCV assay, version 2.0, Roche Molecular Diagnostics, Branchburg, NJ, USA). The lower limit of quantitation (LLOQ) was 1.2 log IU/mL (15 IU/mL) and the lower limit of detection was less than 1.2 log IU/mL.

To evaluate the impact of baseline resistance-associated variants (RAVs), plasma samples for viral resistance assays were collected from all patients at the baseline (day 1). Samples were also collected for resistance testing in patients with virologic failure and HCV RNA level greater than 1000 IU/mL. Samples were evaluated by population sequencing of the NS3/4A and NS5A genes for variants known to confer resistance to either NS3/4A or NS5A inhibitors. The specific loci analyzed included NS3 amino acid positions 36, 54, 55, 56, 80, 107, 122, 132, 155, 156, 158, 168, 170, and 175, and amino acid positions 28, 30, 31, 58, and 93 within the NS5A gene.

Safety assessments included clinical evaluation of adverse events and monitoring of other study parameters, including vital signs, physical examinations, 12-lead electrocardiograms, and standard laboratory safety tests. Events of clinical interest were defined a priori as overdose (intake in excess of the prescribed dose), alanine aminotransferase (ALT) or aspartate aminotransferase (AST) level greater than 500 IU/L not associated with virologic failure, ALT or AST level more than three times the baseline level and more than 100 IU/L not associated with virologic failure, and alkaline phosphatase level more than three times the ULN. Late elevations in ALT/AST level were defined as ALT/AST level elevation to more than five times the ULN occurring after treatment week 4 in patients with ALT/AST level less than or equal to the ULN between treatment weeks 2 and 4.

In both parts, blood samples for pharmacokinetic analysis were collected from all patients. Intensive pharmacokinetic sampling was performed in treatment week 4 for the intensive pharmacokinetic cohorts in both treatment arms of noncirrhotic patients in part 1 (*n* = 29) and in cirrhotic patients in part 2 (*n* = 7). The collection time points in these intensive pharmacokinetic cohorts were day 1 (before the dose and at 0.5, 2, and 4 h after the dose), treatment week 1 (before the dose), treatment week 2 (before the dose),  treatment week 4 (before the dose and at 0.5, 2, 4, 6, 8, and 12 h after the dose), treatment week 6 (before the dose), treatment week 8 (before the dose and anytime after the dose), treatment week 10 (before the dose), and treatment week 12 (anytime after the dose). Plasma EBR and GZR concentrations were determined by a validated high-performance liquid chromatography–tandem mass spectrometry method, with an EBR LLOQ of 0.25 ng/mL (0.283 nM) and a GZR LLOQ of 1.0 ng/mL (1.30 nM).

### Statistical analysis

Approximately 30 patients were to be randomized to each treatment arm in part 1. With this sample size, if the true SVR12 rate was assumed to be 90%, the possibility of observation of an SVR12 rate of less than 75% was less than 1%. If a particular adverse event was not observed in any of the 30 patients in either treatment group in part 1, the true proportion of patients with the adverse event would be less than 8% with 91% confidence.

This study was designed with the hypothesis that the percentage of treatment-naïve patients achieving SVR12 would be greater than 75% in part 2. The reference rate of 75% was taken from the SVR24 rate in treatment-naïve patients receiving telaprevir and peginterferon plus ribavirin in clinical trials in Japan (73%). Of the 240 patients in part 2, the minimum number of patients to be enrolled was set at 140 for treatment-naïve patients and 20 each for prior IFN-based treatment relapsers, nonresponders, and intolerant patients. With use of a 3:1 randomization ratio, 15 patients in each of these cohorts were to be randomized to arm 1 (immediate-treatment arm). Assuming a response rate of 90% (87%), the study had a power of 98% (86%) to detect an SVR12 rate greater than 75%. Allocation of at least 15 patients from each IFN-experienced subpopulation with the assumption of a 90% response rate for each cohort gave the study a probability of 94.5% of observing an SVR12 rate greater than 75%.

We calculated 95% confidence intervals (CIs) for SVR using the Clopper–Pearson method. The lower limit of the 95% Clopper–Pearson exact CI for the SVR12 rate in treatment-naïve patients in the immediate-treatment group was compared with 75%. The same CI constructs were used in the analysis of non-treatment-naïve cohorts.

The full analysis set served as the primary population for efficacy data analysis in both parts and included all randomized patients who received at least one dose of the study medication and had any follow-up efficacy measurement. A supportive analysis was performed with the per-protocol population, which excluded patients for whom there were important protocol deviations that might substantially affect the primary and key secondary efficacy outcomes. Safety analyses were performed in all the patients as the treated population, which comprised all randomized patients who received at least one dose of the study medication.

Analysis of safety during the double-blind period of part 2 was conducted by a tiered approach. Adverse events of special interest that were identified a priori constituted tier 1 events and were subject to inferential testing with *p* values and 95% CIs provided for between-group comparisons. Tier 2 parameters (defined as adverse events occurring in 12 or more patients in arm 1) were analyzed with use of point estimates with 95% CIs provided for between-group comparisons, and tier 3 parameters (all remaining events and parameters) were analyzed with use of point estimates by treatment group. The selection of 12 patients as the threshold for tier 2 was based on the lower limit of the 95% CI difference between arms 1 and 2, in percent incidence, not exceeding zero when the patient numbers were less than 12 in arm 1.

## Results

Part 1 of the study was conducted at 19 trial centers in Japan. It was initiated on August 4, 2014 and was completed on August 28, 2015. Part 2 is being conducted at 50 trial centers across Japan; it was initiated on March 3, 2015 and is ongoing.

### Efficacy

#### Part 1

**Fig. 1:**
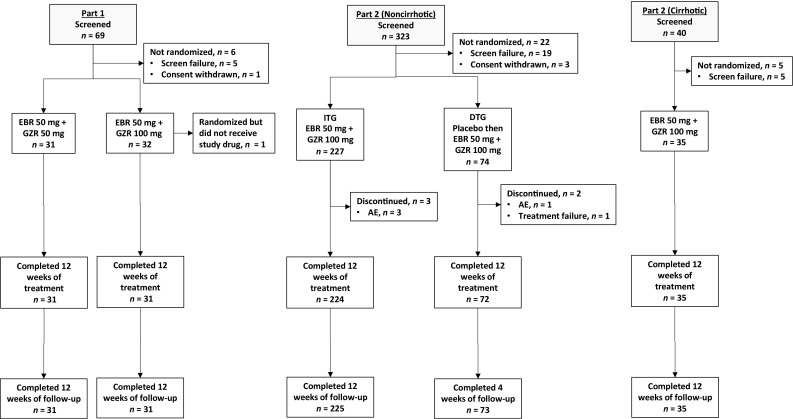
Patient disposition. *AE* adverse event, *DTG* deferred-treatment group, *EBR* elbasvir, *GZR* grazoprevir, *ITG* immediate-treatment group

Sixty-nine patients were screened, and 63 patients were randomized to receive EBR in combination with GZR at a dose of 50 mg (*n* = 31) or 100 mg (*n* = 32) (Fig. 1). One patient (in the 100 mg GZR group) did not receive the study treatment after randomization because of an adverse event during screening. The remaining 62 patients completed the 12-week treatment and 24-week follow-up periods. Baseline characteristics were comparable between treatment arms. Approximately 60% of patients were female, approximately 40% were 65 years or older, and all had HCV GT1b infection. Approximately half of the patients were treatment naïve, 10% were IFN intolerant, and 20% had previously relapsed while receiving IFN-based therapy (Table 1).

**Table 1:** Patient demographics: part 1 and part 2

	Part 1		Part 2		
	EBR + 50 mg GZR (*n* = 31)	EBR + 100 mg GZR (*n* = 32)	ITG: EBR + GZR (*n* = 227)	DTG: placebo (*n* = 74)	Cirrhotic patients (*n* = 35)
Age (years)					
Mean^a^	61.1 (9.7)	58 (12.5)	61.0 (12.5)	60.9 (10.8)	64.8 (9.2)
Median^b^	62 (35–78)	58.5 (30–76)	63.0 (21–80)	63.0 (34–80)	65.0 (43–79)
Sex					
Male	12 (38.7%)	15 (46.9%)	87 (38.3%)	21 (28.4%)	18 (51.4%)
Female	19 (61.3%)	17 (53.1%)	140 (61.7%)	53 (71.6%)	17 (48.6%)
Japanese patients	31 (100%)	32 (100%)	227 (100%)	74 (100%)	35 (100%)
Body mass index, mean (kg/m^2^)^a^	22.8 (3.9)	23 (3.4)	22.7 (3.0)	22.3 (3.5)	23.8 (3.0)
Baseline HCV RNA, geometric mean (log_10_ IU/mL)^a^	6.2 (0.5)	6.2 (0.5)	6.2 (0.5)	6.3 (0.5)	6.2 (0.5)
HCV genotype					
1a	0 (0%)	0 (0%)	4 (1.8%)	1 (1.4%)	1 (2.9%)
1b	31 (100%)	32 (100%)	223 (98.2%)	73 (98.6%)	34 (97.1%)
*IL28B* rs12979860					
Major (CC)	19 (61.3%)	20 (62.5%)	131 (57.7%)	44 (59.5%)	22 (62.9%)
Minor (TC)	12 (38.7%)	10 (31.3%)	86 (37.9%)	29 (39.2%)	13 (37.1%)
Minor (TT)	0 (0%)	1 (3.1%)	10 (4.4%)	1 (1.4%)	0 (0%)
Unknown	0 (0%)	1 (3.1%)	0 (0%)	0 (0%)	0 (0%)
*IL28B* rs8099917					
Major (TT)	19 (61.3%)	21 (65.6%)	136 (59.9%)	46 (62.2%)	24 (68.6%)
Minor (TG)	12 (38.7%)	10 (31.3%)	81 (35.7%)	27 (36.5%)	11 (31.4%)
Unknown	0 (0%)	1 (3.1%)	10 (4.4%)	1 (1.4%)	0 (0%)
Cirrhosis					
No	31 (100%)	31 (96.9%)	227 (100%)	74 (100%)	0 (0%)
Yes	0 (0%)	0 (0%)	0 (0%)	0 (0%)	35 (100%)
Data missing	0 (0%)	1 (3.1%)	0 (0%)	0 (0%)	0 (0%)
HCV treatment history					
Naïve	14 (45.2%)	19 (59.4%)	149 (65.6%)	49 (66.2%)	20 (57.1%)
Intolerant	4 (12.9%)	2 (6.3%)	11 (4.8%)	3 (4.1%)	3 (8.6%)
Relapse	7 (22.6%)	6 (18.8%)	33 (14.5%)	12 (16.2%)	4 (11.4%)
Breakthrough	1 (3.2%)	1 (3.1%)	7 (3.1%)	1 (1.4%)	2 (5.7%)
Partial responder	3 (9.7%)	2 (6.3%)	10 (4.4%)	3 (4.1%)	2 (5.7%)
Null responder	2 (6.5%)	2 (6.3%)	17 (7.5%)	6 (8.1%)	4 (11.4%)
Laboratory values					
Baseline hemoglobin level, mean (g/dL)^a^	13.6 (1.2)	13.8 (1.3)	14.0 (1.5)	13.7 (1.4)	13.6 (1.6)
Baseline platelet count, mean (×10^4^/µL)^a^	19.7 (6.8)	21.2 (6.5)	19.2 (5.8)	19.4 (6.5)	10.6 (3.9)
Baseline ALT level, mean (IU/L)^a^	49 (30.5)	33.8 (12.3)	45.6 (36.5)	41.2 (29.4)	52.6 (24.6)
Baseline AST level, mean (IU/L)^a^	43.6 (20.9)	32.7 (12.5)	43.8 (25.7)	43.5 (30.7)	59.2 (21.7)
Baseline bilirubin level, mean (mg/dL)^a^	0.7 (0.3)	0.5 (0.2)	0.7 (0.3)	0.6 (0.2)	0.8 (0.3)

*ALT* alanine aminotransferase, *AST* aspartate aminotransferase, *DTG* deferred-treatment group, *EBR* elbasvir, *GZR* grazoprevir, *HCV* hepatitis C virus, *ITG* immediate-treatment group

^a^The standard deviation is given in *parentheses*

^b^The range is given in *parentheses*

**Fig. 2:**
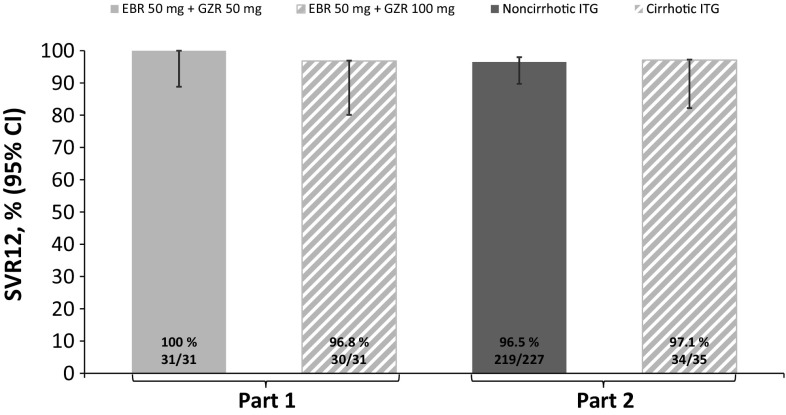
Rate of sustained virologic response at 12 weeks (*SVR12*) in patients receiving elbasvir (*EBR*; 50 mg) plus grazoprevir (*GZR*; 50 or 100 mg) in part 1 and in noncirrhotic and cirrhotic patients receiving EBR at a dose of 50 mg plus GZR at a dose of 100 mg (immediate-treatment group, *ITG*, only) in part 2 (full analysis set). SVR12 rates are not yet available for the deferred-treatment group. *CI* confidence interval

The rates of virologic response were similar between treatment arms. However, virologic response occurred earlier in patients receiving EBR plus GZR at a dose of 100 mg than in those receiving EBR plus GZR at a dose of 50 mg, with very early rapid viral response rates in treatment week 2 of 35.5% (11/31) and 22.6% (7/31) respectively and rapid viral response rates in treatment week 4 of 83.9% (26/31) and 77.4% (24/31) respectively. In all patients, HCV RNA was undetectable by the end of treatment, and the SVR4 rate was 100% (31/31) for both treatment arms. One patient receiving EBR plus GZR at a dose of 100 mg relapsed in follow-up week 12, yielding SVR12 rates of 100% (31/31) in the EBR plus 50 mg GZR arm and 96.8% (30/31) in the EBR plus 100 mg GZR arm (Fig. 2). One patient receiving EBR plus GZR at a dose of 50 mg had a plasma HCV RNA level less than 1.2 log IU/mL in follow-up week 24, but HCV RNA was undetectable 4 weeks later and was therefore considered to have achieved SVR. The SVR24 rate, defined as undetectable HCV RNA in follow-up week 24, was 96.8% (30/31) for both treatment arms.

#### Part 2

Three hundred twenty-three noncirrhotic patients were screened in part 2; 19 failed screening and three withdrew their consent. Of the remaining 301 patients, 227 were randomized to the immediate-treatment group and 74 were randomized to the deferred-treatment group. Five patients (immediate-treatment group, *n* = 3; deferred-treatment group, *n* = 2) failed to complete the initial treatment and the 12-week follow-up period (Fig. 1). Most noncirrhotic patients in part 2 were treatment naïve (66%), and 15% had previously relapsed (Table 1). For the third arm of the study, 40 cirrhotic patients were screened, of whom 35 were enrolled. In this arm, 57% (*n* = 20) were treatment naïve, 11% (*n* = 4) had previously relapsed, and 11% (*n* = 4) had previously had null response.

**Fig. 3:**
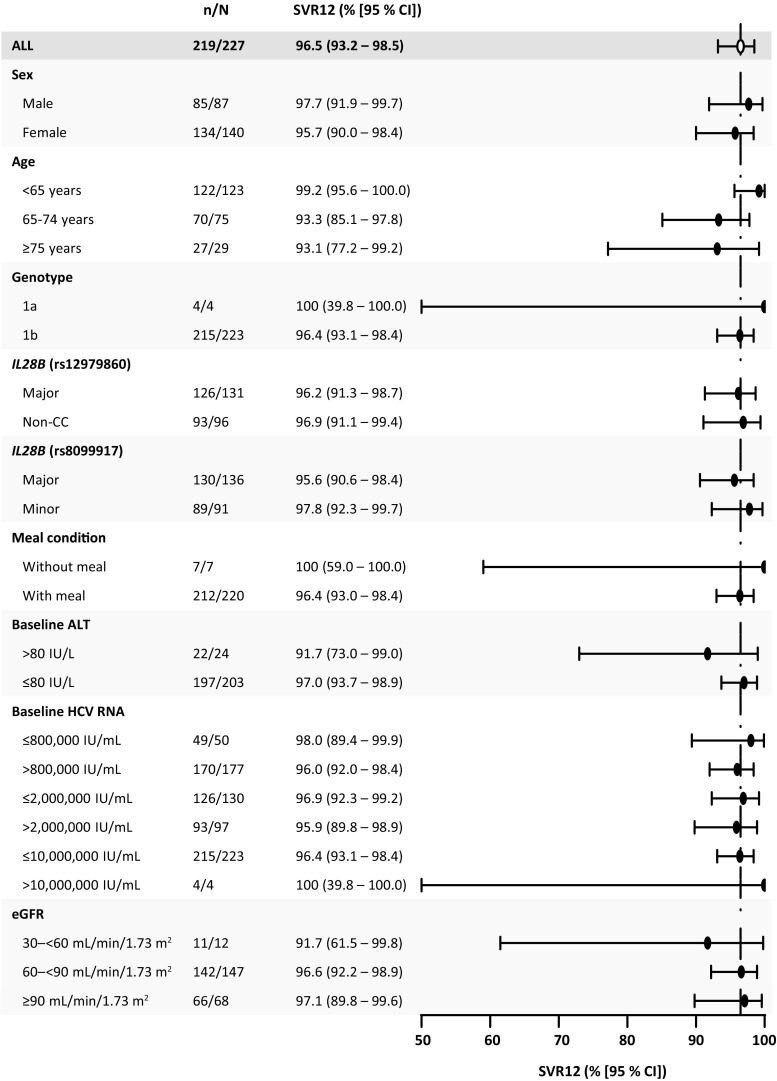
Subgroup analysis of rate of sustained virologic response at 12 weeks (*SVR12*): immediate-treatment group (part 2; full analysis set). *ALT* alanine aminotransferase, *CI* confidence interval, *eGFR* estimated glomerular filtration rate, *HCV* hepatitis C virus

SVR12 was achieved by 219 of 227 patients in the immediate-treatment group (96.5%; 95% CI 93.2–98.5%) (Fig. 2). The lower limit of the 95% CI was higher than the reference rate of 75%, demonstrating that EBR at a dose of 50 mg in combination with GZR at a dose of 100 mg is efficacious. The SVR12 rates were greater than 90% regardless of prior treatment history: the SVR12 rates were 97% (144/149), 91% (10/11), 100% (40/40), and 93% (25/27) in treatment-naïve, IFN-intolerant, prior relapse, and prior nonresponder patients respectively. Eight patients in the full analysis set failed to achieve SVR12: three patients discontinued treatment because of nonvirologic failure (discontinuation due to an adverse event, *n* = 2; discontinuation due to administrative reasons, *n* = 1) and five patients relapsed. No patient had on-treatment virologic breakthrough. In the supportive per-protocol analysis of treatment-naïve patients (excluding patients with missing data), SVR12 was achieved by 142 of 144 patients (98.6%; 95% CI 95.1–99.8%). Subgroup analyses indicated high efficacy across the most important patient subgroups (Fig. 3). SVR12 was achieved in 99% of patients younger than 65 years and 93% of those aged 65 years or older, although it is noteworthy that seven of the eight patients who failed to achieve SVR12 were aged 65 years or older. SVR12 was unaffected by *IL28B* subtype, food administration, or baseline viral load, and all five patients with HCV GT1a infection also achieved SVR12. SVR12 was achieved by 34 of 35 cirrhotic patients (97.1%; 95% CI 85.1–99.9%) and one patient relapsed (Fig. 2).

### Resistance analysis

Three hundred twenty-four patients were eligible for inclusion in the resistance analysis population (62 patients from part 1 and 227 patients from the immediate-treatment group and 35 cirrhotic patients from part 2). Three patients were excluded (two patients discontinued treatment because of serious adverse events and one patient died before follow-up week 12), and the remaining 321 patients were included in the resistance analyses, including seven patients with virologic failure.

NS3 RAVs were detected at the baseline in 103 of the 321 patients (32.1%) (Table S1). Of the 103 patients with baseline RAVs, only five had NS3 RAVs at position 168 (associated with a more than fivefold decrease in GZR potency): two patients had D168E and three patients had D168D/E. All RAVs were detected in patients infected with HCV GT1b. The commonest baseline NS3 RAVs were at S122; however, these RAVs do not reduce susceptibility to GZR (fivefold or less decrease in GZR potency). The Q80K RAV (known to be associated with decreased efficacy of simeprevir plus peginterferon plus ribavirin in GT1a-infected patients) was found in two of five patients (40%) infected with HCV GT1a and two of 316 patients (0.6%) infected with HCV GT1b. SVR12 was achieved by 211 of the 218 patients (96.8%) with no NS3 RAVs at the baseline and all 103 patients (100%) with baseline NS3 RAVs (Table S2).

NS5A RAVs were present at the baseline in 58 of the 321 patients (18.1%) (Table S1). The commonest NS5A RAVs were at Y93 (associated with more than fivefold shift in EBR potency), observed in 43 of the 321 patients (13.4%), including Y93H in 22 patients, Y93Y/H in 19 patients, and Y93Y/C in two patients; only one of these patients was infected with HCV GT1a. SVR12 was achieved by 54 of the 58 patients (93.1%) with NS5A RAVs at the baseline and 260 of the 263 patients (98.9%) without NS5A RAVs at the baseline (Table S2). The SVR rate in patients with baseline Y93 variants was 93.0% (40/43).

Postbaseline resistance analysis was conducted in the seven patients with virologic failure (Table S3). NS3 RAVSs were not detected in any of these seven patients at any time, including at the time of virologic failure. Four relapse patients had baseline NS5A RAVs (Y93H, *n* = 3; L31 M, *n* = 1); three of these four patients acquired treatment-emergent RAVs and had both L31M and Y93H at the time of virologic failure. Of the three patients with wild-type NS5A at the baseline, two had treatment-emergent Y93H and one had both L31M and Y93H.

### Pharmacokinetics

Steady-state plasma exposure of GZR in treatment week 4 was higher in cirrhotic patients than in noncirrhotic patients, with geometric mean ratios for cirrhotic/noncirrhotic patients of 2.16 (90% CI 1.32–3.55) for AUC_0-24 h_, 1.91 (90% CI 1.15–3.15) for *C*_max_, and 2.85 (90% CI 1.62–5.02) for *C*_trough_. Steady-state plasma exposure of GZR in treatment week 4 was higher in patients treated with EBR plus GZR at a dose of 100 mg than in those treated with EBR plus GZR at a dose of 50 mg (geometric mean AUC_0–24 h_ 3.28 µM h vs 1.30 µM h, geometric mean *C*_max_ 0.62 µM vs 0.16 µM, and geometric mean *C*_trough_ 27.78 nM vs 23.43 nM). The median GZR *T*_max_ was approximately 2 h in both cirrhotic and noncirrhotic patients and was not dependent on GZR dose. Steady-state plasma exposures of EBR in treatment week 4 were similar between cirrhotic and noncirrhotic patients [geometric mean ratio for cirrhotic/noncirrhotic patients of 0.95 (90% CI 0.64–1.42) for AUC_0–24 h_, 0.86 (90% CI 0.58–1.28) for *C*_max_, and 1.09 (90% CI 0.67–1.78) for *C*_trough_] and between the EBR plus 50 mg GZR and EBR plus 100 mg GZR treatment arms (geometric mean AUC_0-24 h_ 2.61 µM h vs 2.48 µM h, geometric mean *C*_max_ 0.20 µM vs 0.20 µM, and geometric mean *C*_trough_ 65.70 nM vs 60.89 nM). The median EBR *T*_max_ was also similar between cirrhotic and noncirrhotic patients. The median *T*_max_ was 2.01 h (range 1.92–8.00 h) in patients treated with EBR plus GZR at a dose of 50 mg and 3.95 h (range 1.97–5.98 h) in those treated with EBR plus GZR at a dose of 100 mg; the similar ranges suggest no influence of GZR dose on EBR *T*_max_.

### Safety

#### Part 1

Overall, the frequency and nature of adverse events were similar in patients receiving EBR plus GZR at dose of 50 mg and in patients receiving EBR plus GZR at a dose of 100 mg (Table 2). Drug-related adverse events were reported by ten patients (32.3%) in the 50 mg arm GZR and nine patients (29.0%) in the 100 mg GZR arm. The most commonly reported drug-related adverse event was headache, reported by four patients (12.9%) in the 50 mg GZR arm and three patients (9.7%) in the 100 mg GZR arm. All other drug-related adverse events were reported by less than 5% of patients in both arms. One patient in each arm reported a serious adverse event (acute coronary syndrome in a patient receiving GZR at a dose of 50 mg and hematochezia plus large intestine polyp in a patient receiving GZR at a dose of 100 mg). In addition, one patient receiving GZR at a dose of 50 mg reported a serious adverse event of adenocarcinoma of the colon outside the safety observation window (more than 4 months after the final dose). No patient discontinued treatment because of an adverse event. During the treatment period through follow-up week 4, no patients in either treatment arm had ALT or AST values that met the criteria for late ALT or AST level elevation (more than five times the ULN); one patient in the EBR plus 50 mg GZR arm experienced an increase in ALT level, which met the criteria for a hepatic laboratory event of clinical interest. The event occurred on day 85, and the ALT level returned to within the normal limits approximately 1 month later.

**Table 2:** Safety and adverse events (*AEs*) in part 1 (all patients as treated; initial treatment phase through follow-up week 4)

AEs	50 mg EBR + 50 mg GZR (*n* = 31)	50 mg EBR + 100 mg GZR (*n* = 31)
≥1 AE^a^	21 (67.7%)	23 (74.2%)
Nasopharyngitis	7 (22.6%)	10 (32.3%)
Headache	4 (12.9%)	3 (9.7%)
Pyrexia	3 (9.7%)	1 (3.2%)
Dry eye	2 (6.5%)	0 (0%)
Upper abdominal pain	2 (6.5%)	1 (3.2%)
Diarrhea	2 (6.5%)	1 (3.2%)
Accidental overdose	1 (3.2%)	2 (6.5%)
Drug-related AE	10 (32.3%)	9 (29.0%)
SAE^b^	1 (3.2%)	1 (3.2%)
Discontinuation because of AEs	0 (0%)	0 (0%)
Deaths	0 (0%)	0 (0%)
ALT		
1.1–2.5 times baseline level	0 (0%)	0 (0%)
>2.5–5.0 times baseline level	1 (3.2%)	0 (0%)
>5.0 times baseline level	0 (0%)	0 (0%)
AST		
1.1–2.5 times baseline level	1 (3.2%)	1 (3.2%)
>2.5–5.0 times baseline level	0 (0%)	0 (0%)
>5.0 times baseline level	0 (0%)	0 (0%)
Total bilirubin		
>2.5–5.0 times baseline level	0 (0%)	0 (0%)
>5.0–10.0 times baseline level	0 (0%)	0 (0%)
>10.0 times baseline level	0 (0%)	0 (0%)
Alkaline phosphatase		
1.1–2.5 times baseline level	4 (12.9%)	3 (9.7%)
>2.5–5.0 times baseline level	0 (0%)	0 (0%)
>5.0 times baseline level	0 (0%)	0 (0%)

*ALT* alanine aminotransferase, *AST* aspartate aminotransferase, *EBR* elbasvir, *GZR* grazoprevir, *SAE* serious AE

^a^Incidence greater than 5% in one or more treatment groups

^b^SAEs of acute coronary syndrome in one patient receiving GZR at a dose of 50 mg and hematochezia with large intestine polyp in one patient receiving GZR at a dose of 100 mg. One patient receiving GZR at a dose of 50 mg reported an SAE of adenocarcinoma of the colon outside the safety observation window (more than 4 months after the final dose)

#### Part 2

Eight patients (3.5%) in the immediate-treatment group reported a tier 1 adverse event, compared with no patients in the deferred-treatment group (Table 3). The treatment difference was 3.5% (95% CI −1.5 to 6.8 with *p* = 0.102), indicating that the incidence of tier 1 events did not differ significantly between the treatment arms. All eight events were elevated laboratory values reported as events of clinical interest. Four of the patients with a tier 1 event of clinical interest in the immediate-treatment group met the criteria for late elevation of ALT/AST level to more than five times the ULN. These events were observed in treatment week 8 for one patient, in treatment week 10 for one patient, and in treatment week 12 for two patients. No patient in the deferred-treatment group had an elevation to more than five times the ULN. The tier 2 adverse events (adverse events occurring in 12 or more patients in either treatment arm) were nasopharyngitis and increased ALT level. There was no statistical difference in the incidence of these adverse events between treatment arms [15% vs 16.2% for nasopharyngitis, difference −1.2 percentage points (95% CI −12.1 to 7.4 percentage points); 5.7% vs 1.4% for increased ALT level, difference 4.4 percentage points (95% CI −1.8 to 8.5 percentage points)].

**Table 3:** Safety and adverse events (*AEs*) in noncirrhotic patients enrolled in part 2 (all patients as treated; initial treatment phase through follow-up week 4)

AEs	ITG: EBR + GZR (*n* = 227)	DTG: placebo (*n* = 74)	Percentage point difference^f^
Tier 1 (events of clinical interest)	8 (3.5%)	0 (0%)	3.5 (−1.5 to 6.8); *p* = 0.102
≥1 AEs	147 (64.8%)	50 (67.6%)	−2.8 (−14.5 to 10.0)
Nasopharyngitis^a^	34 (15.0%)	12 (16.2%)	−1.2 (−12.1 to 7.4)
ALT level increased^a^	13 (5.7%)	1 (1.4%)	4.4 (−1.8 to 8.5)
Drug-related AE^a^	58 (25.6%)	14 (18.9%)	
ALT level increased	12 (5.3%)	1 (1.4%)	
SAEs^b^	11 (4.8%)	1 (1.4%)	
Drug-related SAEs^c^	2 (1%)	0 (0%)	
Discontinuation because of AEs^d^	3 (1.3%)	1 (1.4%)	
Deaths^e^	0 (0.0%)	0 (0.0%)	
ALT			
1.1–2.5 times baseline level	6 (2.6%)	29 (39.2%)	
>2.5–5.0 times baseline level	6 (2.6%)	0 (0%)	
>5.0 times baseline level	5 (2.2%)	1 (1.4%)	
AST			
1.1–2.5 times baseline level	8 (3.5%)	26 (35.1%)	
>2.5–5.0 times baseline level	6 (2.6%)	0 (0%)	
>5.0 times baseline level	2 (0.9%)	0 (0%)	
Total bilirubin			
>2.5–5.0 times baseline level	1 (0.4%)	0 (0%)	
>5.0–10.0 times baseline level	0 (0%)	0 (0%)	
>10.0 times baseline level	0 (0%)	0 (0%)	
Alkaline phosphatase			
1.1–2.5 times baseline level	32 (14.1%)	9 (12.2%)	
>2.5–5.0times baseline level	0 (0%)	0 (0%)	
>5.0 times baseline level	0 (0%)	0 (0%)	

*ALT* alanine aminotransferase, *AST* aspartate aminotransferase, *DTG* deferred-treatment group, *EBR* elbasvir, *GZR* grazoprevir, *ITG* immediate-treatment group, *SAE* serious AE

^a^Tier 2 adverse events occurring in 12 or more patients in the ITG

^b^Eleven patients (4.8%) in the ITG experienced a total of 14 SAEs and one patient (1.4%) in the DTG had an SAE. Cataract was the only SAE that was reported for more than one patient (two patients in the ITG)

^c^Two patients in the ITG reported drug-related SAEs (cerebral infarction, *n* = 1; increased ALT/AST level, *n* = 1)

^d^The AEs resulting in discontinuation were cardiac sarcoidosis, cerebral infarction, and increased ALT/AST level in the ITG, and hepatocellular carcinoma in the DTG (increased ALT/AST level occurred in a single patient). All of these events were reported as SAEs. Two patients who experienced cardiac sarcoidosis or cerebral infarction in the ITG discontinued treatment in treatment week 2 and did not achieve sustained virologic response. Another patient in the ITG who experienced an ALT/AST level elevation discontinued treatment on day 51 of the study therapy but achieved sustained virologic response at 12 weeks

^e^One patient in the ITG died outsidethe safety observation window (11 weeks after the final dose); the investigator reported that the cause of death was unknown

^f^The 95% confidence interval is given in *parentheses*

Serious adverse events were reported in 11 patients (4.8%) in the immediate-treatment group and in one patient (1.4%) in the deferred-treatment group. Cataract was the only serious adverse event reported in more than one patient (reported by two patients in the immediate-treatment group). Two serious adverse events in the immediate-treatment group were considered to be related to the study drug by the investigator (cerebral infarction and increased ALT/AST level). Both patients with drug-related serious adverse events discontinued treatment with the study drug. A third patient, with cardiac sarcoidosis, in the immediate-treatment group also discontinued treatment, and one patient in the deferred-treatment group discontinued treatment because of hepatocellular carcinoma. Other than the events of clinical interest described above, the elevations in AST/ALT level were generally mild and decreased from the baseline with continued therapy. There were no total bilirubin level elevations to more than 5.0 times the baseline level or alkaline phosphatase level elevations to more than 2.5 times the baseline level in either treatment arm.

Late ALT/AST level elevations to more than five times the ULN occurred in 2 of 34 patients with cirrhosis (5.9%) and met the criteria for events of clinical interest. These late ALT/AST level elevations were observed in treatment weeks 10 and 12, and were reported to have been resolved on discontinuation of treatment by follow-up week 4. Late ALT/AST level elevations for both patients were accompanied by slight increases in bilirubin level (to approximately 1.3 times the ULN) and eosinophil level (approximately 6.3%), but not in the international normalized ratio. In addition, one other patient had elevated ALT levels in follow-up week 4 of therapy that met the criteria for classification as an event of clinical interest. Among the patients with cirrhosis, there were 13 patients with reported drug-related adverse events (Table 4), the commonest of which (reported in more than 5% of patients) were increased ALT level (14.3%, 5/35), increased AST level (14.3%, 5/35), diarrhea (8.6%, 3/35), constipation (5.7%, 2/35), and malaise (5.7%, 2/35). There were no serious adverse events, and no cirrhotic patient discontinued treatment early because of an adverse event.

**Table 4:** Safety and adverse events (*AEs*) in cirrhotic patients enrolled in part 2 (all patients as treated; initial treatment phase through follow-up week 4)

AEs	Cirrhotic patients (*n* = 35)
Events of clinical interest	3 (8.6%)
≥1 AEs^a^	28 (80%)
Nasopharyngitis	5 (14.3%)
Increased ALT level	5 (14.3%)
Increased AST level	5 (14.3%)
Rash	3 (8.6%)
Constipation	3 (8.6%)
Diarrhea	3 (8.6%)
Malaise	2 (5.7%)
Anemia	2 (5.7%)
Headache	2 (5.7%)
Drug-related AEs^b^	13 (37.1%)
SAEs	0 (0%)
Discontinuation because of AEs	0 (0%)
Deaths	0 (0%)
ALT	
1.1–2.5 times baseline level	2 (5.7%)
>2.5–5.0 times baseline level	2 (5.7%)
>5.0 times baseline level	1 (2.9%)
AST	
1.1–2.5 times baseline level	3 (8.6%)
>2.5–5.0 times baseline level	1 (2.9%)
>5.0 times baseline level	1 (2.9%)
Total bilirubin	
>2.5–5.0 times baseline level	0 (0%)
>5.0–10.0 times baseline level	0 (0%)
>10.0× baseline	0 (0%)
Alkaline phosphatase	
1.1–2.5 times baseline level	7 (20%)
>2.5–5.0 times baseline level	0 (0%)
>5.0 times baseline level	0 (0%)

*ALT* alanine aminotransferase, *AST* aspartate aminotransferase, *SAEs* serious AEs

^a^Incidence greater than 5%

^b^All mild intensity. The drug-related AEs most commonly reported for 5% of patients were increased ALT level (14.3%, 5/35), increased AST level (14.3%, 5/35), diarrhea (8.6%, 3/35), constipation (5.7%, 2/35), and malaise (5.7%, 2/35)

## Discussion

This study demonstrated that the once daily oral combination regimen of 50 mg EBR and 100 mg GZR, given for 12 weeks, is highly efficacious and well tolerated in cirrhotic and noncirrhotic Japanese patients with chronic HCV infection. In the phase II study, GZR doses of 50 and 100 mg were similarly effective, with SVR rates of 100% and 96.8% respectively, and only one patient in the 100 mg GZR arm experienced virologic relapse (there were no instances of virologic breakthrough in the phase II study). Although there is no clear evidence that early virologic response is predictive of SVR12 among patients receiving regimens consisting of only direct-acting antiviral agents, the proportion of patients with undetectable HCV RNA in treatment week 2 was slightly higher in the 100 mg GZR group than the 50 mg GZR group [35.5% (95% CI 19.2–54.6%) vs 22.6% (95% CI 9.6–41.1%)]. Tolerability was similar in both arms, with a comparable incidence of drug-related adverse events (32.3% vs 29.0%), and no patients in either treatment arm discontinued treatment because of an adverse event or had ALT or AST values that met the criteria for late ALT or AST level elevation.

On the basis of the higher response rate in treatment week 2 and the absence of late AST and ALT level elevations (which have been reported at higher doses of GZR) [16], coupled with the generally comparable safety profile of both GZR doses, it was decided that the 100 mg GZR dose would be evaluated further in combination with EBR at a dose of 50 mg in the phase III study, consistent with the dosing regimen evaluated in phase III studies in Western patients [11, 14, 15].

In the phase III study, 96.5% of noncirrhotic patients in the immediate-treatment group and 97.1% of cirrhotic patients achieved SVR12, supporting the use of this treatment regimen in Japanese patients with chronic HCV infection. Eight noncirrhotic patients and one cirrhotic patient failed to achieve SVR12; six relapsed and three failed to achieve virologic response (no patient had on-treatment virologic breakthrough). Among noncirrhotic patients, efficacy was similar in patients older than 65 years and patients aged 75 years or older (93% in both groups), and was slightly lower than in patients younger than 65 years (99.2%). Seven of eight noncirrhotic patients who failed to achieve SVR12 were older than 65 years. Across the entire study population, the SVR12 rates were also high in patients with and without NS3 RAVs at the baseline (100% vs 96.8%) and with and without NS5A RAVs (93.1% vs 98.9%). All seven patients who relapsed had NS5A RAVs at the time of relapse. Safety was also generally similar between the immediate-treatment group and the deferred-treatment group. Eight tier 1 events were reported in patients receiving EBR plus GZR, all of which were elevated laboratory values reported as events of clinical interest and four of which met the criteria for late elevation of ALT/AST level to more than five times the ULN. Late ALT/AST level elevations to more than five times the ULN also occurred in two patients (5.9%) with cirrhosis. Overall, the incidence of tier 1 and tier 2 events did not differ significantly between the noncirrhotic patients in the randomized immediate-treatment group and deferred-treatment group.

The findings from this study are broadly similar to those from the phase II/III evaluation of EBR plus GZR in Western patients. These studies show high efficacy (more than 90%) across a broad cross section of patients, and in particular show consistently high response rates among patients with cirrhosis [9–12, 14, 15]. Analysis of data from these studies shows that the most impactful variable in predicting SVR12 in patients receiving EBR plus GZR is the presence of NS5A RAVs in patients with HCV GT1a infection. Patients with HCV GT1a infection and baseline NS5A RAVs require treatment with EBR and GZR plus ribavirin for 16 weeks. In patients with HCV GT1b infection, treatment with EBR plus GZR for 12 weeks is recommended for all patients except for those with prior failure of regimen containing a direct-acting antiviral agent, in which case the addition of ribavirin is recommended [6]. In the present study, 98% of enrolled patients had HCV GT1b infection; overall, HCV GT1a infection is rare in Japan [1]. The SVR12 rate remained more than 92% in patients infected with HCV GT1b with baseline NS5A RAVs, although a slightly lower response rate was seen in patients with baseline NS5A RAVs than in patients without NS5A RAVs (92.9% vs 98.8%). Of the seven patients infected with HCV GT1b with virologic failure, four had L31 and Y93 double mutations.

Several other all-oral direct-acting antiviral agent regimens have been evaluated in Japanese patients with HCV infection. A 12-week regimen of ledipasvir plus sofosbuvir resulted in SVR12 rates of 100% in 171 treatment-naïve and treatment-experienced patients primarily infected with HCV GT1b . Similarly to the present study, the SVR12 rates were unaffected by the presence of cirrhosis or baseline NS5A RAVs [17]. In a phase III, 12-week study of ombitasvir plus paritaprevir plus ritonavir, the SVR12 rates were 94.9% (204/215) in Japanese noncirrhotic patients and 90.5% (38/42) in Japanese cirrhotic patients. The overall virologic failure rate in that study was 3% (11/363): eight patients relapsed and three had on-treatment virologic failure [18]. In that study, the Y93H NS5A variant was present at the baseline in 8 of 11 patients with virologic failure. This variant is reported to be present in 21.4% of Japanese patients with HCV GT1b infection [19], and is also associated with decreased activity to daclatasvir, which is approved for use in Japanese patients without this polymorphism [5, 20]. In Japanese patients with HCV GT1b infection receiving daclatasvir once daily and asunaprevir twice daily for 24 weeks, SVR24 was achieved by 87.4% (118/135) of IFN-intolerant or ineligible patients and 80.5% (70/87) of patients with prior nonresponse. The SVR24 rate in cirrhotic patients was 90.9% (20/22), and 15% of patients in that study had virologic failure (34/222; breakthrough, *n* = 14; nonresponder, *n* = 3; relapse, *n* = 17) [20].

In conclusion, data from the present study indicate that treatment with EBR plus GZR for 12 weeks is effective and well tolerated in patients with HCV GT1b infection. In the phase III part of this study, SVR12 was achieved in 96.5% of patients, with high efficacy maintained in the important patient subgroups aged 65 years or older and with baseline NS5A RAVs. EBR plus GZR therefore represents a safe and effective treatment option for Japanese patients with HCV GT1b infection.

## Electronic supplementary material

Below is the link to the electronic supplementary material.

Supplementary material 1 (PDF 351 kb)

Supplementary material 2 (PDF 398 kb)

Supplementary material 3 (PDF 342 kb)
