# Supplementary material for: The combination of elbasvir and grazoprevir for the treatment of chronic HCV infection in Japanese patients: a randomized phase II/III study
Source: J Gastroenterol. 2016 Nov 21;52(4):520–33. doi: 10.1007/s00535-016-1285-y (PMC5357479; doi:10.1007/s00535-016-1285-y)
Supplement: Supplementary file 1 — Supplementary material 1 (PDF 351 kb) [file 535_2016_1285_MOESM1_ESM.pdf]

Electronic Supplemental Material for *The Journal of Gastroenterology*

The Combination of Elbasvir and Grazoprevir for the Treatment of Chronic HCV Infection in Japanese Patients: A Randomized Phase II/III Study

Hiromitsu Kumada · Yoshiyuki Suzuki · Yoshiyasu Karino · Kazuaki Chayama · Norifumi Kawada · Takeshi Okanoue · Yoshito Itoh · Satoshi Mochida · Hidenori Toyoda · Hitoshi Yoshiji · Shintaro Takaki · Naoyoshi Yatsuzuka · Etsuo Yodoya · Takashi Iwasa · Go Fujimoto · Michael N. Robertson · Stuart Black · Luzelena Caro · Janice Wahl

Corresponding Author: Naoyoshi Yatsuzuka, naoyoshi_yatsuzuka@merck.com

Table S1 Prevalence of baseline NS3 and NS5A RAVs in parts 1 and 2

Population	Total number of patients	Number of patients with sequences available	Number of patients With RAVs		Baseline RAVs (number of patients with variants detected)
			RAVs not detected, *n* (%)	RAVs detected, *n* (%)	
**NS3 RAVs**					
Overall	321	321	218 (67.9)	103 (32.1)	D168D/E (3), D168E (2), M175L (2), M175L/M (1), Q80K (4), S122G (57), S122S/G (13), S122T/A (1), S122T/S/A/G (1), T54S (12), T54S/T (1), V107I (1), V107I/V (1), V158I (1), V170I/M/V (2), V170I/T (1), V170I/V (8), V36L (1), V55A (1)
**By genotype and subtype**					
GT1a	5	5	3 (60.0)	2 (40.0)	Q80K (2)
GT1b	316	316	215 (68.0)	101 (32.0)	D168D/E (3), D168E (2), M175L (2), M175L/M (1), Q80K (2), S122G (57), S122S/G (13), S122T/A (1), S122T/S/A/G (1), T54S (12), T54S/T (1), V107I (1),

					V107I/V (1), V158I (1), V170I/M/V (2), V170I/T (1), V170I/V (8), V36L (1), V55A (1)
**NS5A RAVs**					
Overall	321	321	263 (81.9)	58 (18.1)	L31L/I/M (1), L31L/M (1), L31M (11), L31V (1), M28M/V (1), R30H (1), R30Q/R (1), Y93H (22), Y93Y/C (2), Y93Y/H (19)
**By genotype and subtype**					
GT1a	5	5	3 (60.0)	2 (40.0)	M28M/V (1), Y93Y/C (1)
GT1b	316	316	260 (82.3)	56 (17.7)	L31L/I/M (1), L31L/M (1), L31M (11), L31V (1), R30H (1), R30Q/R (1), Y93H (22), Y93Y/C (1), Y93Y/H (19)

RAVs resistance-associated variants, *GT*, genotype
