# Supplementary material for: The combination of elbasvir and grazoprevir for the treatment of chronic HCV infection in Japanese patients: a randomized phase II/III study
Source: J Gastroenterol. 2016 Nov 21;52(4):520–33. doi: 10.1007/s00535-016-1285-y (PMC5357479; doi:10.1007/s00535-016-1285-y)
Supplement: Supplementary file 2 — Supplementary material 2 (PDF 398 kb) [file 535_2016_1285_MOESM2_ESM.pdf]

The Combination of Elbasvir and Grazoprevir for the Treatment of Chronic HCV Infection in Japanese Patients: A Randomized Phase II/III Study

Hiromitsu Kumada · Yoshiyuki Suzuki · Yoshiyasu Karino · Kazuaki Chayama · Norifumi Kawada · Takeshi Okanoue · Yoshito Itoh · Satoshi Mochida · Hidenori Toyoda · Hitoshi Yoshiji · Shintaro Takaki · Naoyoshi Yatsuzuka · Etsuo Yodoya · Takashi Iwasa · Go Fujimoto · Michael N. Robertson · Stuart Black · Luzelena Caro · Janice Wahl

Corresponding Author: Naoyoshi Yatsuzuka, naoyoshi_yatsuzuka@merck.com

Table S2 Impact of baseline NS3 and NS5A RAVs on SVR12 in parts 1 and 2

Population	SVR12, *n/N* (%)		
	Overall efficacy in patients with sequence	RAVs not detected	RAVs detected
**NS3 RAVs**			
Overall	314/321 (97.8)	211/218 (96.8)	103/103 (100.0)
**By genotype and subtype**			
GT1a	5/5 (100.0)	3/3 (100.0)	2/2 (100.0)
GT1b	309/316 (97.8)	208/215 (96.7)	101/101 (100.0)
**NS5A RAVs**			
Overall	314/321 (97.8)	260/263 (98.9)	54/58 (93.1)

**By genotype and subtype**			
GT1a	5/5 (100.0)	3/3 (100.0)	2/2 (100.0)
GT1b	309/316 (97.8)	257/260 (98.8)	52/56 (92.9)

RAVs resistance-associated variants, *GT*, genotype
