# Supplementary material for: The combination of elbasvir and grazoprevir for the treatment of chronic HCV infection in Japanese patients: a randomized phase II/III study
Source: J Gastroenterol. 2016 Nov 21;52(4):520–33. doi: 10.1007/s00535-016-1285-y (PMC5357479; doi:10.1007/s00535-016-1285-y)
Supplement: Supplementary file 3 — Supplementary material 3 (PDF 342 kb) [file 535_2016_1285_MOESM3_ESM.pdf]

The Combination of Elbasvir and Grazoprevir for the Treatment of Chronic HCV Infection in Japanese Patients: A Randomized Phase II/III Study

Hiromitsu Kumada · Yoshiyuki Suzuki · Yoshiyasu Karino · Kazuaki Chayama · Norifumi Kawada · Takeshi Okanoue · Yoshito Itoh · Satoshi Mochida · Hidenori Toyoda · Hitoshi Yoshiji · Shintaro Takaki · Naoyoshi Yatsuzuka · Etsuo Yodoya · Takashi Iwasa · Go Fujimoto · Michael N. Robertson · Stuart Black · Luzelena Caro · Janice Wahl

Corresponding Author: Naoyoshi Yatsuzuka, naoyoshi_yatsuzuka@merck.com

Table S3 RAVs in 7 patients with virologic failure

		Day 1		At failure	
Patient	GT	NS3	NS5A	NS3	NS5A
110182	1b	WT	***Y93Y/H***	WT	L31M, ***Y93H***
105810	1b	WT	WT	WT	Y93H
105330	1b	WT	***L31M***	WT	***L31M,*** Y93H
110313	1b	WT	WT	WT	Y93H[a]
105135	1b	WT	***Y93H***	WT	***Y93H***
105804	1b	WT	***Y93H***	WT	L31M, ***Y93H***
105136	1b	WT	WT	WT	L31M, Y93H

GT genotype, *HCV* hepatitis C virus, *RAVs* resistance-associated variants, *WT* wild-type

[a] At time of failure (follow-up week 4), HCV RNA was insufficient (<3 log IU/mL) for RAV analysis in this patient. At follow up week 12, HCV RNA was sufficient to detect Y93H
